# Frailty Predicts Mortality and Procedural Performance in Patients With Non‐Variceal Upper Gastrointestinal Bleeding

**DOI:** 10.1002/jgh3.70188

**Published:** 2025-05-21

**Authors:** Ali Jaan, Adeena Maryyum, Hassam Ali, Umer Farooq, Dushyant Singh Dahiya, Qurat Ul Ain Muhammad, Fernando J. Castro

**Affiliations:** ^1^ Division of Internal Medicine Unity Hospital Rochester New York USA; ^2^ Division of Internal Medicine Ayub Medical College Abbottabad Pakistan; ^3^ Division of Gastroenterology East Carolina University Greenville North Carolina USA; ^4^ Division of Gastroenterology Saint Louis University Saint Louis Missouri USA; ^5^ Division of Gastroenterology University of Kansas School of Medicine Kansas City Kansas USA; ^6^ Division of Internal Medicine Rawalpindi Medical University Rawalpindi Pakistan; ^7^ Division of Gastroenterology Cleveland Clinic Florida Weston Florida USA

**Keywords:** endoscopy, frailty, gastrointestinal hemorrhage, hospitalization, nonvariceal upper gastrointestinal bleeding, risk assessment

## Abstract

**Introduction:**

Nonvariceal upper gastrointestinal bleeding (NVUGIB) is a common cause of hospitalization in the United States, with approximately 400 000 admissions annually and a 5%–10% mortality rate. This study aimed to evaluate frailty's impact on NVUGIB outcomes.

**Methods:**

We utilized the 2019 National Readmission Database (NRD) to identify adult patients (≥ 18 years) admitted with a principal diagnosis of NVUGIB using ICD‐10‐CM codes. NVUGIB hospitalizations were stratified by frailty using the hospital frailty risk score (HFRS) of 5 or more as the cut‐off for frailty. Multivariate regression analyses were conducted to analyze the outcomes. STATA 14.2 was used for statistical testing.

**Results:**

Among 218 647 NVUGIB admissions, 99 892 (45.69%) were frail. Frail patients were older, more often female, and had higher comorbidity burdens. They showed significantly greater in‐hospital mortality (adjusted odds ratio [aOR] 5.64, 95% CI 4.94–6.44; *p* < 0.001), acute kidney injury (5.85), respiratory failure (6.93), septic shock (40.94), hemorrhagic shock (2.64), vasopressor use (4.36), mechanical ventilation (6.04), and ICU admission (5.41). Although frail patients had higher odds of esophagogastroduodenoscopy (EGD) with intervention (1.04; *p* < 0.001), they were less likely to receive EGD within 24 h (0.75; *p* < 0.001). They also had higher odds of rebleeding (1.18; *p* < 0.001) and radioembolization (2.69; *p* < 0.001). Length of stay increased by 2.30 days, total charges rose by $28 518, discharge to rehabilitation was more frequent (3.12; *p* < 0.01), and 30‐day readmission was higher (15.24% vs. 11.43%, HR 1.16; *p* < 0.001).

**Conclusion:**

Frailty independently predicts worse clinical outcomes and increased resource use in NVUGIB. Recognizing frailty may improve risk stratification and guide more tailored management strategies for this high‐risk population.

## 1 Introduction

Bleeding from the gastrointestinal (GI) tract, proximal to the ligament of Treitz, in the absence of esophageal, gastric, or duodenal varices is termed non‐variceal upper gastrointestinal bleeding (NVUGIB) [1]. Peptic ulcer disease is known to be the most common cause of NVUGIB [2]. The impact of NVUGIB on the healthcare system in the United States is significant, as evidenced by the hospitalization rate of 67 per 100 000 individuals and a mortality rate of 5%–10%, translating to approximately 400 000 admissions and costs exceeding one billion dollars yearly [3, 4, 5, 6].

Frailty is a complex multidimensional syndrome characterized by diminished physiological reserves across different organ systems, reducing the body's capacity to withstand stressors such as acute illness or injury [6, 7]. Commonly used tools to identify frail patients include the frailty phenotype model, the five‐factor modified frailty index (mFI‐5), and the hospital frailty risk score (HFRS) [8, 9, 10]. Frailty has emerged as a significant health determinant that leads to poor hospitalization outcomes, including increased mortality rates [11]. Established risk prediction models, such as the Glasgow Blatchford Scale (GBS) and the Rockall score, are widely used in the context of upper gastrointestinal bleeding (UGIB) [12, 13]. Although frailty has been extensively studied as a predictor of adverse outcomes in cardiovascular and pulmonary diseases, its role in NVUGIB remains largely underexplored [8, 14]. Only a few small‐scale studies have attempted to assess the predictive utility of frailty in NVUGIB outcomes [15].

Given the dearth of large‐scale data exploring the interplay between frailty and NVUGIB, this study aims to bridge this gap. Our objective is to examine the relationship between frailty status and clinical outcomes in NVUGIB patients, as well as its influence on the interventions performed and readmission rates.

## 2 Methods

### 2.1 Database Information

Our research involved a retrospective cohort analysis using the Nationwide Readmission Database (NRD) for the years 2016–2020 [16]. The database is compiled from a 20% stratified sample of all U.S. community hospitals, excluding rehabilitation and long‐term care facilities. Each discharge from the resultant data is then weighted to ensure national representativeness. When weighted, the NRD data represents an estimated 32 million hospitalizations across the country. This comprehensive database contains over 100 de‐identified clinical and non‐clinical elements for each hospital stay at both hospital and patient levels.

### 2.2 Study Population

We included adult patients (aged ≥ 18 years) admitted with a principal diagnosis of NVUGIB utilizing the International Classification of Diseases, Tenth Revision, and Clinical Modifications (ICD‐10‐CM) codes where the principal diagnosis was recorded in the DX1 field. To accurately identify hospitalized patients with NVUGIB, we reviewed previously published literature on NVUGIB [17]. The ICD‐10‐CM diagnosis and procedure codes used in our study are listed in Table S1. Gilbert et al. [10] created and validated the HFRS using 109 ICD‐10‐CM codes, which were found to be present disproportionately in frail patients. Each diagnosis code was assigned a unique value based on its predictive potential for frailty, with higher values indicating a stronger predictive power (Table S2). An HFRS of 5 or above was used to classify the patients as frail [10]. We used the same HFRS cutoff to classify a patient as frail in our study.

Although our study utilized the NRD database, which contains de‐identified data, approval was sought from the institutional review board. Given the nature of the data, this study was deemed exempt from full review.

### 2.3 Study Variables and Outcomes

The primary outcome assessed was the influence of frailty on inpatient mortality. Secondary outcomes evaluated included acute kidney injury (AKI), need for blood transfusion, acute respiratory failure, hemorrhagic shock, septic shock, vasopressor requirement, need for mechanical ventilation, and intensive care unit (ICU) admission. Additionally, we compared the severity of bleeding among the frail and non‐frail cohorts. Since numerical values were not available in the dataset utilized, we devised surrogate NVUGIB severity scores using diagnostic codes corresponding to individual components of AIMS‐65 (comprising Albumin, International normalized ratio (INR), Mental status, Systolic blood pressure, and age 65 or older) and Pre‐endoscopic Rockall scores [18]. These scores were calculated by summing the points assigned to each component, as defined by the AIMS‐65 and Pre‐endoscopic Rockall score criteria. Furthermore, procedural performance, including esophagogastroduodenoscopy (EGD) and interventional radiology (IR)‐guided embolization, was compared between groups. Lastly, we examined resource utilization with measures including 30‐day readmission rate, total hospitalization charges (THC), length of stay (LOS), and proportion of patients discharged to rehabilitation services among both groups.

### 2.4 Statistical Analysis

Prior to applying descriptive statistics, the normality of individual variables was assessed through the Kolmogorov–Smirnov test. The continuous variables, specifically length of stay (LOS) and total hospitalization charges, followed a Poisson distribution. Consequently, we employed the Mann–Whitney *U* test to assess means and Poisson regression to assess the adjusted mean difference in the linear regression model. Categorical variables were analyzed using the Pearson *χ* ^2^ test, and binary outcomes were determined using logistic regression analysis. In our survival analysis, we considered the time from discharge to readmission as the time variable and readmission as failure. The unadjusted hazard ratio (HR) for 30‐day readmission was computed using univariable Cox regression analysis. We conducted multivariate regression analyses incorporating confounding variables with a *p*‐value ≤ 0.2 from univariate regression analysis [19]. We also included variables deemed clinically significant to the outcome, regardless of their statistical significance, in the univariate analysis. Final variables included in the regression analysis were: age, gender, Charlson comorbidity index (CCI), insurance status (Medicare, Medicaid, private, and uninsured), hospital bed size (small, medium, and large), hospital location (rural vs. rural), and hospital teaching status. All *p* values were two‐sided, with statistical significance defined as *p* less than 0.05. We performed our analyses using STATA, version 14.2 (StataCorp., College Station, Texas, USA).

## 3 Results

### 3.1 Patient Characteristics and Comorbid Status

**FIGURE 1:**
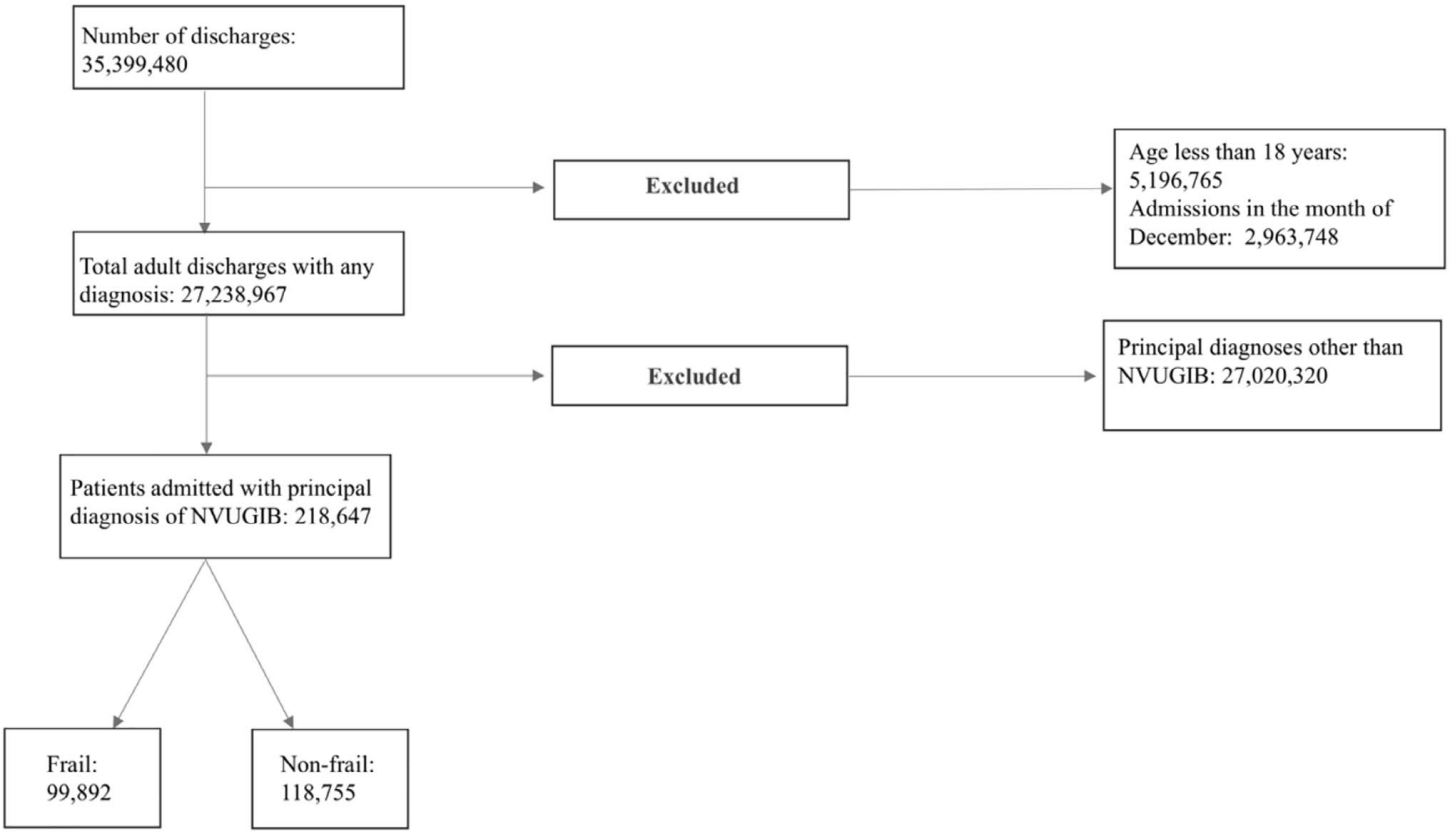
Flowsheet for patient selection for the current study.

A total of 218 647 adult patients admitted with NVUGIB were identified, of whom 99 892 (45.69%) were frail (HFRS ≥ 5) (Figure 1). Table 1 presents their demographic and hospital characteristics. Frail patients were notably older, more often female, and had higher Charlson Comorbidity Index (CCI) scores (≥ 3). Regarding insurance status, a greater proportion of frail patients were covered by Medicare. Table 2 outlines comorbidity distribution among frail and non‐frail patients, showing a higher prevalence of congestive heart failure, end‐stage renal disease, myocardial infarction, cerebrovascular disease, peripheral vascular disease, chronic obstructive pulmonary disease, and diabetes in the frail group.

**TABLE 1:** Demographics of non‐variceal upper gastrointestinal bleeding hospitalizations, stratified by frailty status.

Baseline characteristics	NVUGIB + Age ≥ 18: (*n* = 218,647)		
	Non‐frail (*n* = 118,755)	Frail (*n* = 99,892)	*p*
Mean age (years)	65.30	70.85	< 0.001
Female gender, %	45.24	48.02	< 0.001
Charlson comorbidity index, %			< 0.001
0	8.31	3.31	
1	34.24	17.57	
2	28.76	25.56	
≥ 3	28.69	53.56	
Median household income in the patient's zip code (quartile), %			0.796
1st (0–25th)	29.55	29.79	
2nd (26–50th)	26.49	26.43	
3rd (51st–75th)	25.33	25.01	
4th (76–100th)	18.64	18.77	
Insurance status, %			< 0.001
Medicare	60.46	75.43	
Medicaid	12.78	9.82	
Private	21.77	12.19	
Uninsured	4.98	2.56	
Hospital bed size, %			0.478
Small	18.52	17.99	
Medium	28.2	28.59	
Large	53.28	53.43	
Hospital teaching status, %			< 0.001
Non‐teaching	28.07	26.27	
Teaching	71.93	73.73	
Hospital location, %			0.290
Rural	6.45	6.15	
Urban	93.55	93.85	

Abbreviation: NVUGIB, nonvariceal upper gastrointestinal bleeding.

**TABLE 2:** Comorbid status of non‐variceal upper gastrointestinal bleeding hospitalizations, stratified by frailty status.

Comorbidities	NVUGIB + Age ≥ 18: (*n* = 218,647)		
	Non‐frail (*n* = 118,755)	Frail (*n* = 99,892)	*p*
Congestive heart failure, %	18.68	30.58	< 0.001
End‐stage renal disease, %	16.73	41.05	< 0.001
History of myocardial infarction, %	9.86	12.65	< 0.001
History of cerebrovascular disease, %	1.56	9.88	< 0.001
Peripheral vascular disease, %	9.03	12.42	< 0.001
Chronic obstructive pulmonary disease, %	20.61	27.07	< 0.001
Diabetes, %	13.05	24.75	< 0.001
Rheumatoid arthritis, %	3.15	3.85	< 0.001
Peptic ulcer disease, %	63.45	63.78	0.328
Liver cirrhosis, %	8.08	8.50	0.026
Hemiplegia/paraplegia	0.26	1.42	< 0.001

### 3.2 Mortality and Morbidity

In‐hospital mortality for NVUGIB was significantly higher in frail patients compared to non‐frail counterparts (adjusted odds ratio [aOR] 5.64, 95% confidence interval [CI] 4.94–6.44, *p* < 0.001) (Table 3). Frailty was also associated with higher odds of acute kidney injury (aOR 5.85, *p* < 0.001), acute respiratory failure (aOR 6.93, *p* < 0.001), septic shock (aOR 40.94, *p* < 0.001), hemorrhagic shock (aOR 2.64, *p* < 0.001), vasopressor use (aOR 4.36, *p* < 0.001), mechanical ventilation (aOR 6.04, *p* < 0.001), and ICU admission (aOR 5.41, *p* < 0.001). The likelihood of requiring blood transfusion was also higher in frail patients (aOR 1.11, *p* < 0.001). Frail patients exhibited significantly higher proportions of adverse clinical variables, including hypoalbuminemia, abnormal INR, altered mental status, and comorbid conditions, leading to elevated mean severity scores for AIMS‐65 (0.88 vs. 0.65) and Pre‐endoscopic Rockall (3.64 vs. 2.46) compared to non‐frail patients (*p* < 0.001) (Table 4).

**TABLE 3:** Unadjusted and adjusted outcomes of non‐variceal upper gastrointestinal bleeding hospitalizations, stratified by frailty status.

Outcomes	NVUGIB + Age ≥ 18: (*n* = 218,647)			
	Non‐frail (*n* = 118,755)	Frail (*n* = 99,892)	Adjusted OR (95% CI)	*p*
Mortality, %	0.46	3.21	5.64 (4.94–6.44)	< 0.001
AKI, %	8.88	40.19	5.85 (5.63–6.07)	< 0.001
Blood transfusion, %	35.10	40.47	1.11 (1.08–1.16)	< 0.001
Acute respiratory failure, %	1.28	9.32	6.93 (6.34–7.57)	< 0.001
Septic shock, %	0.03	1.37	40.94 (25.43–65.91)	< 0.001
Hemorrhagic shock, %	1.80	4.92	2.64 (2.43–2.87)	< 0.001
Vasopressor requirement, %	0.38	1.91	4.36 (3.58–5.30)	< 0.001
Mechanical ventilation, %	1.17	6.74	6.04 (5.47–6.67)	< 0.001
Requiring ICU admission, %	1.47	7.67	5.41 (4.91–5.96)	< 0.001

Abbreviations: AKI, acute kidney injury; CI, confidence interval; ICU, intensive care unit; ICU, intensive care unit; NVUGIB, non‐variceal upper gastrointestinal bleeding; OR, odds ratio.

**TABLE 4:** Surrogate severity score for non‐variceal upper gastrointestinal bleeding hospitalizations, derived from component variables of the AIMS‐65 and pre‐endoscopic Rockall scores.

NVUGIB + Age ≥ 18: (*n* = 218 647)			
Variables	Non‐frail (*n* = 118,755)	Frail (*n* = 99,892)	*p*
AIMS‐65 score components (points per AIMS‐65 score)			
Hypoalbuminemia (1 point), %	0.91	2.05	< 0.001
Abnormal INR (1 point), %	3.36	5.97	< 0.001
Altered mental status (1 point), %	0.16	2.03	< 0.001
Hemorrhagic shock (1 point), %	1.94	5.45	< 0.001
Age ≥ 65 years (1 point), %	58.08	72.01	< 0.001
Surrogate severity score^a^ (per AIMS‐65 score criteria), mean	0.65	0.88	< 0.001
Pre‐endoscopic Rockall score components (points per pre‐endoscopic Rockall score)			
Age group			< 0.001
< 60 years (0 point), %	33.10	20.19	
60–79 years (1 point), %	47.52	49.40	
≥ 80 years (2 points), %	19.38	30.41	
Hemodynamic status			< 0.001
Tachycardia (1 point), %	2.65	3.80	
Hypotension/shock (2 points), %	8.57	24.17	
Comorbidities			< 0.001
Heart failure, ischemic heart disease or cerebrovascular disease (2 points), %	24.57	22.96	
Renal failure, hepatic failure or disseminated cancer (3 points), %	30.30	51.92	
Surrogate severity score^a^ (per pre‐endoscopic Rockall score criteria), mean	2.46	3.64	< 0.001

Abbreviation: INR, international normalized ratio.

^a^ Calculated by adding points assigned to each component, as defined by the respective score criteria.

### 3.3 Procedural Performance

**FIGURE 2:**
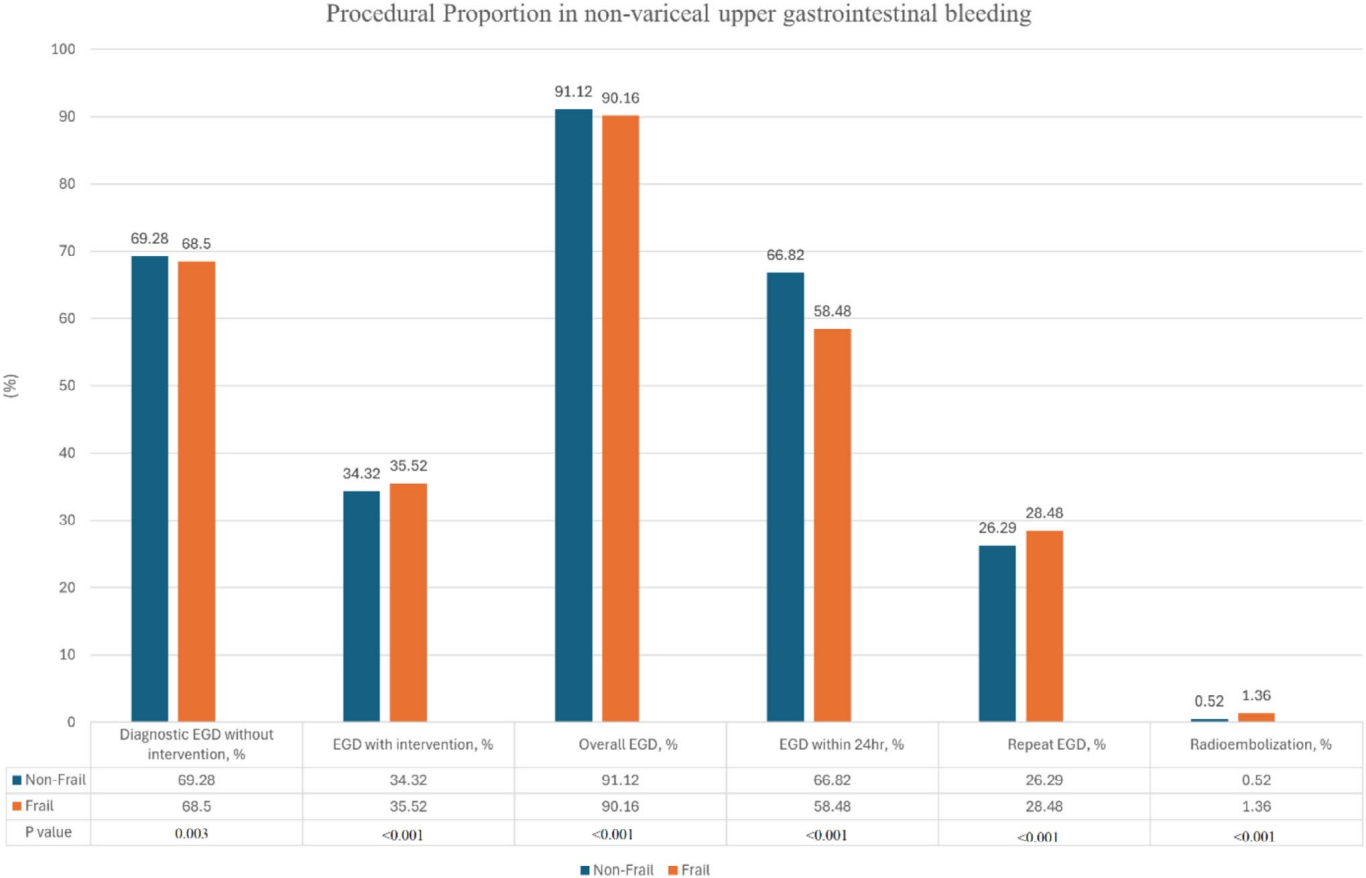
Procedural proportion in non‐variceal upper gastrointestinal bleeding based on frailty.

Frail patients had higher odds of undergoing EGD with intervention (AOR 1.04, *p* < 0.001) but lower odds of EGD performed within 24 h (AOR 0.75, *p* < 0.001) and overall EGD (AOR 0.82, *p* < 0.001) (Table 5, Figure 2). Rebleeding, assessed by the need for repeat EGD within the same hospitalization, was found to have higher odds in frail patients (AOR 1.18, *p* < 0.001). Finally, the odds of requiring radioembolization were also higher in frail patients (AOR 2.69, *p* < 0.001).

**TABLE 5:** Procedural proportion in non‐variceal upper gastrointestinal bleeding hospitalizations, stratified by frailty status.

Outcomes	NVUGIB + Age ≥ 18: (*n* = 218,647)			
	Non‐frail (*n* = 118,755)	Frail (*n* = 99,892)	Adjusted OR (95% CI)	*p*
Diagnostic EGD, %	69.28	68.5	0.93 (0.90–0.95)	0.003
EGD with intervention, %	34.32	35.52	1.04 (1.02–1.08)	< 0.001
Overall EGD, %	91.12	90.16	0.82 (0.79–0.87)	< 0.001
EGD within 24 h, %	66.82	58.48	0.75 (0.73–0.77)	< 0.001
Repeat EGD, %	26.29	28.48	1.18 (1.14–1.21)	< 0.001
Radiologic embolization, %	0.52	1.36	2.69 (2.30–3.14)	< 0.001

Abbreviations: CI, confidence interval; EGD, esophagogastroduodenoscopy; NVUGIB, non‐variceal upper gastrointestinal bleeding; OR, odds ratio.

### 3.4 Resource Utilization

**FIGURE 3:**
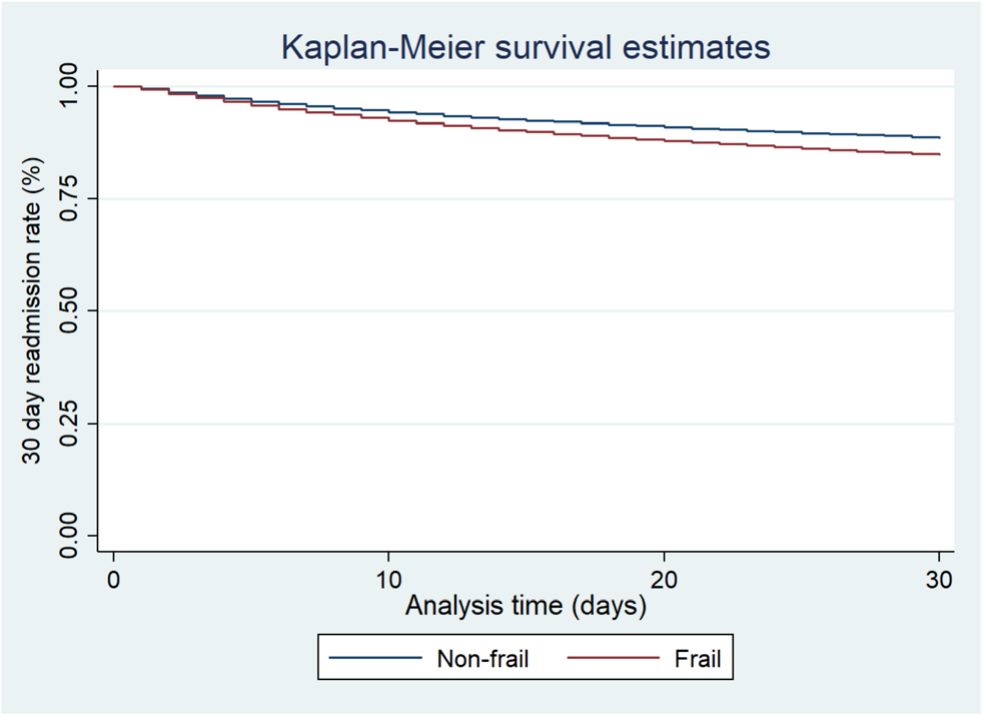
Kaplan–Meier curve in non‐variceal upper gastrointestinal bleeding based on frailty.

Frail patients had increased mean LOS by 2.30 days (*p* < 0.001), a significantly higher THC by a mean of $28 518 (*p* < 0.001), and were more likely to be discharged to rehabilitation facilities (AOR 3.12, *p* < 0.001) (Table 6). Additionally, the 30‐day readmission rate was 15.24% for frail patients, while it was 11.43% for non‐frail counterparts with an adjusted HR of 1.16 (*p* < 0.001). Figure 3 depicts the Kaplan–Meier curve for 30‐day readmission. Analysis of 30‐day readmission for NVUGIB also revealed elevated rates in frail patients (3.89 vs. 3.49, *p* < 0.001) with an adjusted HR of 1.07 (*p* = 0.041).

**TABLE 6:** Resource utilization in non‐variceal upper gastrointestinal bleeding hospitalizations, stratified by frailty status.

Outcomes	NVUGIB + Age ≥ 18: (*n* = 218,647)			
	Non‐frail (*n* = 118,755)	Frail (*n* = 99,892)	Adjusted MD (95% CI)	*p*
Mean LOS, days (95% CI)	3.43 (3.38–3.47)	6.12 (6.03–6.21)	2.30 (2.22–2.37)	< 0.001
Mean THC, USD (95% CI)	42 448 (41 198–43 699)	74 523 (72 057–76 989)	28 518 (27 042–29 995)	< 0.001
Rehabilitation discharge, %	7.68	25.44	3.12^a^ (2.99–3.26)	< 0.001
30‐day readmission, %	11.43	15.24	1.16^b^ (1.11–1.21)	< 0.001
30‐day readmission for NVUGIB, %	3.49	3.89	1.07^b^ (1.03–1.11)	0.041

Abbreviations: CI, confidence interval; LOS, length of hospital stay; MD, mean difference; NVUGIB, non‐variceal upper gastrointestinal bleeding; THC, total hospitalization charges; USD, United States dollar.

^a^ Adjusted odds ratio.

^b^ Hazard ratio.

## 4 Discussion

Using nationally representative data, our cohort identified frailty in 45.69% of individuals with NVUGIB, with a higher prevalence among the elderly, females, and those with elevated CCI scores. This distribution closely aligns with patterns reported in the existing literature [20, 21]. Moreover, frailty was significantly associated with poor clinical outcomes, including heightened mortality and morbidity, increased likelihood of repeat endoscopy, and need for radiological intervention. These patients also utilized greater healthcare resources, as evidenced by prolonged hospital stays, higher costs, higher 30‐day readmission, and elevated 30‐day rebleeding rates.

Frailty has been widely reported to be associated with increased mortality [22], likely attributable to the diminished functional homeostatic reserve impairing the body's ability to manage physiological and pathological stressors effectively [23]. Guliyara et al. [15] in a single‐center, prospective cohort study, reported a two‐fold increased mortality risk among frail patients. Our study corroborates this elevated risk by demonstrating almost five‐fold higher mortality rates among frail NVUGIB patients. Additionally, frailty is associated with significantly greater odds of complications—such as AKI, acute respiratory failure, and septic and hemorrhagic shock—that are both independently triggered and exacerbated by frailty. This trend is well documented across a range of other diseases [24, 25, 26, 27].

While the impact of frailty on mortality and periprocedural complications in GI bleeding patients is established, its role in predicting morbidity and resource utilization remains debated [15, 28]. Guliyara et al., based on a cohort of 298 adults with NVUGIB, did not find any significant association between frailty and resource use. In contrast, our study highlights frailty as a powerful predictor of critical outcomes, including increased ICU admission, extended hospital stays, higher 30‐day readmission, and elevated 30‐day rebleeding rates. Several factors might account for the differing results between our study and that of Guliyara et al. One key difference is the smaller sample size used by Guliyara et al., which may limit the detection of subtle yet clinically important associations. In contrast, our study utilizes a larger national dataset from NRD, enhancing statistical power, which may amplify the measurable impact of frailty on utilization outcomes. Second, the use of different frailty measurement scales, with Guliyara et al. employing the Canadian Study of Health and Aging Clinical Frailty Scale (CSHA‐CFS) and our study using the HFRS, could also explain the variation in findings. Additionally, the differences in healthcare practices, resource availability, or admission criteria between study settings could affect resource utilization outcomes. Together, these differences suggest that prior insignificant findings may reflect limitations in sensitivity and power rather than the absence of a true relationship. Our results advocate for the routine integration of comprehensive frailty measures into NVUGIB risk models, emphasizing frailty's broader implications for clinical outcomes and healthcare resource allocation.

The likelihood of performing procedural interventions, such as EGD, in frail patients was significantly lower, particularly within the first 24 h. This reduction in overall EGD may be attributed to the potential biases in decision‐making due to increased periprocedural complications in this population, as reported by Acosta et al. [28]. They concluded that a positive frailty status was associated with a two times greater likelihood of periprocedural complications [28]. However, it could be argued that the reduced utilization of EGD may result in delays that could negatively impact patient outcomes [29]. Nonetheless, the increased likelihood of requiring repeat EGD and radioembolization within this cohort highlights the probable occurrence of persistent or recurrent bleeding, a notion supported by the significantly higher frequency of blood transfusions needed by frail patients. This concept is further reinforced by comparing these trends to those observed in other studies, where extensive literature supports the association between frailty and rebleeding [15, 30, 31]. To the best of our knowledge, this study represents the first national‐level analysis to investigate the impact of frailty on the performance of NVUGIB‐related procedural interventions.

Frailty's role as a predictor for ICU admission [32] is likely due to the complex interplay of physiological vulnerability, existing comorbidities, and severity of complications within this cohort [33]. The longer LOS and higher 30‐day readmission rates underscore the profound healthcare challenges encountered by the frail population, echoing the patterns documented in previous studies [34]. The strong association between frailty and NVUGIB outcomes noted in our study highlights the necessity for integrating frailty assessment into clinical practice. Identifying frailty risk early can help tailor management strategies, optimize resource allocation, and potentially improve outcomes for this vulnerable population. Additionally, healthcare providers should be aware of the increased risks associated with frail patients and consider early and aggressive interventions, when feasible, to mitigate the likelihood of complications and poor outcomes.

Leveraging the NRD, which captures data from a wide spectrum of hospitals across 32 U.S. states, our study achieves enhanced external validity and generalizability, thereby mitigating biases inherent to single‐ or multi‐center investigations [35]. Furthermore, we incorporated a range of socioeconomic and institutional variables often neglected in institution‐specific studies, further enriching the robustness of our findings. Despite the rigorous methodological approach employed, our study is subject to some limitations, primarily stemming from the use of a population database. Specifically, variables such as vital signs and laboratory values are not reported in the NRD. Consequently, we were unable to risk‐stratify patients according to the severity of GI bleeding using previously validated scores such as the Glasgow Blatchford Score (GBS), AIMS65, or the Rockall score. To address this limitation, we developed qualitative surrogate severity scores and applied them to our study population. It is also important to mention that frailty was analyzed as a binary variable (frail vs. non‐frail) rather than stratified by severity (e.g., mild, moderate, and severe). This simplification reduces the granularity of outcome prediction and may obscure clinically meaningful distinctions between subgroups of frail patients, as mildly and severely frail patients differ significantly. Lastly, despite the use of multivariate regression analysis to adjust for potential confounding factors, the possibility of residual confounding cannot be entirely excluded.

## 5 Conclusion

This study highlights frailty as a significant predictor of adverse outcomes and increased resource utilization in NVUGIB‐related hospitalizations. Frailty was not only independently associated with higher inpatient mortality, complications, and healthcare use but also influenced procedural decisions. Recognizing frailty in this context may enhance risk stratification and support more individualized management. Future studies should focus on validating frailty assessment tools and integrating them with existing risk scores to improve clinical decision‐making.

## Ethics Statement

Institutional IRB approval was not required for this study as it involved de‐identified data with no direct interaction with human subjects or identifiable personal data.

## Consent

The authors have nothing to report.

## Conflicts of Interest

The authors declare no conflicts of interest.

## Supporting information


**Table S1.** International Classification of Diseases, tenth revision (ICD‐10) codes used for the study.
**Table S2.** 109 International Classification of Diseases, tenth revision (ICD‐10)‐codes and number of points for each diagnosis to create the Hospital Frailty Risk Score (HFRS) in patients admitted for *Clostridium difficile* infection.
