# Supplementary material for: Frailty Predicts Mortality and Procedural Performance in Patients With Non‐Variceal Upper Gastrointestinal Bleeding
Source: JGH Open. 2025 May 21;9(5):e70188. doi: 10.1002/jgh3.70188 (PMC12093336; doi:10.1002/jgh3.70188)
Supplement: Supplementary file 1 — Table S1. International Classification of Diseases, tenth revision (ICD‐10) codes used for the study. Table S2. 109 International Classification of Diseases, tenth revision (ICD‐10)‐codes and number of points for each diagnosis to create the Hospital Frailty Risk Score (HFRS) in patients admitted for Clostridium difficile infection. [file JGH3-9-e70188-s001.docx]

**Table 1S: International Classification of Diseases, tenth revision (ICD-10) codes used for the study.**

| **Disease/Procedure** | **ICD-10-CM codes** |
| --- | --- |
| Nonvariceal upper gastrointestinal bleeding (NVUGIB) | K20.81, K20.91, K21.01, K22.11, K25.0, K25.2, K25.4, K25.6, K26.2, K26.4, K26.6, K27.0, K27.2, K27.4, K27.6, K28.0, K28.2, K28.4, K28.6, K29.01, K29.21, K29.31, K29.41, K29.51, K29.61, K29.71, K29.81, K29.91, K31.811, K31.82 |
| Hemorrhagic/hypovolemic shock | R57.1 |
| Septic shock | R65.21 |
| Acute kidney injury | N17.0, N17.1, N17.2, N17.8, N17.9 |
| Blood transfusion | 30233N1, 30233P1, 30243N1, 30243P1, 30243H1, 30233H1 |
| Acute respiratory failure | J96.0x |
| Vasopressor requirement | 3E063XZ, 3E060XZ, 3E053XZ, 3E050XZ, 3E043XZ, 3E040XZ, 3E033XZ, 3E030XZ |
| Intubation/Mechanical ventilation | 5A0945Z, 5A1935Z, 5A1945Z, 5A1955Z, 0BH17EZ, 0BH18EZ |
| Esophagogastroduodenoscopy (EGD)-Diagnostic | 0DJ08ZZ, 0DJ68ZZ, 0DB18ZX, 0DB18ZZ, 0DB28ZX, 0DB28ZZ, 0DB38ZX, 0DB38ZZ, 0DB48ZX, 0DB48ZZ, 0DB58ZX, 0DB58ZZ, 0DB68ZX, 0DB68ZZ, 0DB78ZX, 0DB78ZZ, 0DB88ZX, 0DB88ZZ, 0DB98ZX, 0DB98ZZ |
| Esophagogastroduodenoscopy (EGD) with intervention | 0D518ZZ, 0D528ZZ, 0D538ZZ, 0D548ZZ, 0D558ZZ, 0D568ZZ, 0D578ZZ, 0D598ZZ, 0DQ18ZZ, 0DQ28ZZ, 0DQ38ZZ, 0DQ48ZZ, 0DQ58ZZ, 0DQ68ZZ, 0DQ78ZZ, 0DQ98ZZ, 0W3P8ZZ, 3E0G8TZ, 06L38CZ |
| Radioembolization (of celiac, super mesenteric, gastric, or splenic artery) | 04L13DZ, 04L23DZ, 04L43DZ, 04L53DZ |

**Table 2S: 109 International Classification of Diseases, tenth revision (ICD-10)-codes and number of points for each diagnosis to create the Hospital Frailty Risk Score (HFRS) in patients admitted for Clostridium difficile infection.**

| **ICD-10 code** | **Diagnosis** | **Points contributed to HRFS** |
| --- | --- | --- |
| F00 | Dementia in Alzheimer | 7·1 |
| G81 | Hemiplegia | 4·4 |
| G30 | Alzheimer's disease | 4·0 |
| I69 | Sequelae of cerebrovascular disease (secondary codes) | 3·7 |
| R29 | Other symptoms and signs involving the nervous and musculoskeletal systems (R29·6 Tendency to fall) | 3·6 |
| N39 | Other disorders of urinary system (includes urinary tract infection and urinary incontinence) | 3·2 |
| F05 | Delirium, not induced by alcohol and other psychoactive substances | 3·2 |
| W19 | Unspecified fall | 3·2 |
| S00 | Superficial injury of head | 3·2 |
| R31 | Unspecified hematuria | 3·0 |
| B96 | Other bacterial agents as the cause of diseases classified to other chapters (secondary code) | 2·9 |
| R41 | Other symptoms and signs involving cognitive functions and awareness | 2·7 |
| R26 | Abnormalities of gait and mobility | 2·6 |
| I67 | Other cerebrovascular diseases | 2·6 |
| R56 | Convulsions, not elsewhere classified | 2·6 |
| R40 | Somnolence, stupor and coma | 2·5 |
| T83 | Complications of genitourinary prosthetic devices, implants and grafts | 2·4 |
| S06 | Intracranial injury | 2·4 |
| S42 | Fracture of shoulder and upper arm | 2·3 |
| E87 | Other disorders of fluid, electrolyte and acid-base balance | 2·3 |
| M25 | Other joint disorders, not elsewhere classified | 2·3 |
| E86 | Volume depletion | 2·3 |
| R54 | Senility | 2·2 |
| F03 | Unspecified dementia | 2·1 |
| W18 | Other fall on same level | 2·1 |
| Z50 | Care involving use of rehabilitation procedures | 2.1 |
| Z75 | Problems related to medical facilities and other health care | 2·0 |
| F01 | Vascular dementia | 2·0 |
| S80 | Superficial injury of lower leg | 2·0 |
| L03 | Cellulitis | 2·0 |
| H54 | Blindness and low vision | 1·9 |
| E53 | Deficiency of other B group vitamins | 1·9 |
| Z60 | Problems related to social environment | 1·8 |
| G20 | Parkinson's disease | 1·8 |
| R55 | Syncope and collapse | 1·8 |
| S22 | Fracture of rib(s), sternum and thoracic spine | 1·8 |
| K59 | Other functional intestinal disorders | 1·8 |
| N17 | Acute renal failure | 1·8 |
| L89 | Decubitus ulcer | 1·7 |
| Z22 | Carrier of infectious disease | 1·7 |
| B95 | Streptococcus and staphylococcus as the cause of diseases classified to other chapters | 1·7 |
| L97 | Ulcer of lower limb, not elsewhere classified | 1.6 |
| R44 | Other symptoms and signs involving general sensations and perceptions | 1·6 |
| K26 | Duodenal ulcer | 1·6 |
| I95 | Hypotension | 1·6 |
| N19 | Unspecified renal failure | 1·6 |
| A41 | Other septicemia | 1·6 |
| Z87 | Personal history of other diseases and conditions | 1·5 |
| J96 | Respiratory failure, not elsewhere classified | 1·5 |
| X59 | Exposure unspecified factor | 1·5 |
| M19 | Other arthrosis | 1·5 |
| G40 | Epilepsy | 1·5 |
| M81 | Osteoporosis without pathological fracture | 1·4 |
| S72 | Fracture of femur | 1·4 |
| S32 | Fracture of lumbar spine and pelvis | 1·4 |
| E16 | Other disorders of pancreatic internal secretion | 1·4 |
| R94 | Abnormal results of function studies | 1·4 |
| N18 | Chronic renal failure | 1·4 |
| R33 | Retention of urine | 1·3 |
| R69 | Unknown and unspecified causes of morbidity | 1·3 |
| N28 | Other disorders of kidney and ureter, not elsewhere classified | 1·3 |
| R32 | Unspecified urinary incontinence | 1·2 |
| G31 | Other degenerative diseases of nervous system, not elsewhere classified | 1·2 |
| Y95 | Nosocomial condition | 1·2 |
| S09 | Other and unspecified injuries of head | 1·2 |
| R45 | Symptoms and signs involving emotional state | 1·2 |
| G45 | Transient cerebral ischemic attacks and related syndromes | 1·2 |
| Z74 | Problems related to care-provider dependency | 1·1 |
| M79 | Other soft tissue disorders, not elsewhere classified | 1·1 |
| W06 | Fall involving bed | 1.1 |
| S01 | Open wound of head | 1.1 |
| A04 | Other bacterial intestinal infections | 1·1 |
| A09 | Diarrhea and gastroenteritis of presumed infectious origin | 1.1 |
| J18 | Pneumonia, organism unspecified | 1·1 |
| J69 | Pneumonitis due to solids and liquids | 1·0 |
| R47 | Speech disturbances, not elsewhere classified | 1·0 |
| E55 | Vitamin D deficiency | 1·0 |
| Z93 | Artificial opening status | 1·0 |
| R02 | Gangrene not classified anywhere | 1·0 |
| R63 | Symptoms and signs concerning food and fluid intake | 0·9 |
| H91 | Other hearing loss | 0·9 |
| W10 | Fall on and from stairs and steps | 0·9 |
| W01 | Fall on same level from slipping, tripping and stumbling | 0·9 |
| E05 | Thyrotoxicosis [hyperthyroidism] | 0·9 |
| M41 | Scoliosis | 0·9 |
| R13 | Dysphagia | 0·8 |
| Z99 | Dependence on enabling machines and devices | 0·8 |
| U80 | Agent resistant to penicillin and related antibiotics | 0·8 |
| M80 | Osteoporosis with pathological fracture | 0·8 |
| K92 | Other diseases of digestive system | 0·8 |
| I63 | Cerebral Infarction | 0·8 |
| N20 | Calculus of kidney and ureter | 0·7 |
| F10 | Mental and behavioral disorders due to use of alcohol | 0·7 |
| Y84 | Other medical procedures as the cause of abnormal reaction of the patient | 0·7 |
| R00 | Abnormalities of heart beat | 0·7 |
| J22 | Unspecified acute lower respiratory infection | 0·7 |
| Z73 | Problems related to life-management difficulty | 0·6 |
| R79 | Other abnormal findings of blood chemistry | 0·6 |
| Z91 | Personal history of risk-factors, not elsewhere classified | 0·5 |
| S51 | Open wound of forearm | 0·5 |
| F32 | Depressive episode | 0·5 |
| M48 | Spinal stenosis (secondary code only) | 0·5 |
| E83 | Disorders of mineral metabolism | 0·4 |
| M15 | Polyarthrosis | 0·4 |
| D64 | Other anemias | 0·4 |
| L08 | Other local infections of skin and subcutaneous tissue | 0·4 |
| R11 | Nausea and vomiting | 0·3 |
| K52 | Other noninfective gastroenteritis and colitis | 0·3 |
| R50 | Fever of unknown origin | 0·1 |
